# Association of Conventional Cardiovascular Risk Factors With Cardiovascular Disease After Hypertensive Disorders of Pregnancy: Analysis of the Nord-Trøndelag Health Study

**DOI:** 10.1001/jamacardio.2019.1746

**Published:** 2019-06-12

**Authors:** Eirin B. Haug, Julie Horn, Amanda R. Markovitz, Abigail Fraser, Bjørnar Klykken, Håvard Dalen, Lars J. Vatten, Pål R. Romundstad, Janet W. Rich-Edwards, Bjørn O. Åsvold

**Affiliations:** 1 K.G. Jebsen Center for Genetic Epidemiology, Department of Public Health and Nursing, Norwegian University of Science and Technology, Trondheim, Norway; 2 Department of Public Health and Nursing, Norwegian University of Science and Technology, Trondheim, Norway; 3 Department of Obstetrics and Gynecology, Levanger Hospital, Nord-Trøndelag Hospital Trust, Levanger, Norway; 4 Department of Epidemiology, Harvard T. H. Chan School of Public Health, Harvard University, Boston, Massachusetts; 5 Division of Women’s Health, Department of Medicine, Brigham and Women’s Hospital and Harvard Medical School, Boston, Massachusetts; 6 Mathematica Policy Research, Cambridge, Massachusetts; 7 Department of Population Health Sciences, Bristol Medical School and Medical Research Council Integrative Epidemiology Unit at the University of Bristol, Bristol, England; 8 Department of Medicine, Levanger Hospital, Nord-Trøndelag Hospital Trust, Levanger, Norway; 9 Department of Circulation and Medical Imaging, Norwegian University of Science and Technology, Trondheim, Norway; 10 Cardiac Clinic, St Olavs Hospital, Trondheim University Hospital, Trondheim, Norway; 11 Department of Endocrinology, St Olavs Hospital, Trondheim University Hospital, Trondheim, Norway

## Abstract

**Importance:**

Women with a history of hypertensive disorders of pregnancy (HDP) have higher risk of cardiovascular disease (CVD). It is not known how much of the excess CVD risk in women with a history of HDP is associated with conventional cardiovascular risk factors.

**Objective:**

To quantify the excess risk of CVD in women with a history of HDP and estimate the proportion associated with conventional cardiovascular risk factors.

**Design, Setting, and Participants:**

Prospective cohort study with a median follow-up of 18 years. Population-based cohort of women participating in the Nord-Trøndelag Health Study in Norway. We linked data for 31 364 women from the Nord-Trøndelag Health Study (1984-2008) to validated hospital records (1987-2015), the Cause of Death Registry (1984-2015), and the Medical Birth Registry of Norway (1967-2012). A total of 7399 women were excluded based on selected pregnancy characteristics, incomplete data, or because of emigrating or experiencing the end point before start of follow-up, leaving 23 885 women for study. Data were analyzed between January 1, 2018, and June 6, 2018.

**Exposures:**

Experiencing 1 or more pregnancies complicated by HDP before age 40 years vs only experiencing normotensive pregnancies.

**Main Outcomes and Measures:**

We used Cox proportional hazards models to estimate the hazard ratios (HRs) for the association between HDP and CVD. The proportion of excess risk associated with conventional cardiovascular risk factors was estimated using an inverse odds ratio weighting approach.

**Results:**

Our study population consisted of 23 885 parous women from Nord-Trøndelag County, Norway. A total of 21 766 women had only normotensive pregnancies, while 2199 women experienced ever having an HDP. From age 40 to 70 years, women with history of HDP had an increased risk of CVD compared with women with only normotensive pregnancies (HR, 1.57; 95% CI, 1.32-1.87) but not at older age (β = 0.98; 95% CI, 0.96-1.00; *P* for interaction by age = .01). Blood pressure and body mass index were associated with up to 77% of the excess risk of CVD in women with history of HDP, while glucose and lipid levels were associated with smaller proportions.

**Conclusion and Relevance:**

In this study, the risk of excess CVD in women with history of HDP was associated with conventional cardiovascular risk factors, indicating that these risk factors are important targets for cardiovascular prevention in these women.

## Key Points

**Question:**

Women with history of hypertensive pregnancy disorders have a higher risk of cardiovascular disease, but how much of their excess cardiovascular risk is associated with conventional cardiovascular risk factors?

**Findings:**

In this cohort study, blood pressure and body mass index were associated with up to 77% of the excess risk of cardiovascular disease in women with history of hypertensive pregnancy disorders, while glucose and lipids were associated with smaller proportions.

**Meaning:**

The association of conventional risk factors, in particular blood pressure and body mass index, with the development of cardiovascular in women with history of hypertensive pregnancy disorders indicate that preventive efforts aimed at decreasing the levels of these risk factors could reduce cardiovascular risk in women with history of hypertensive pregnancy disorders.

## Teaser

This cohort study examines the excess risk of cardiovascular disease in women with a history of hypertensive disorders of pregnancy and estimates the proportion associated with conventional cardiovascular risk factors.

## Introduction

Women with history of hypertensive disorders of pregnancy (HDP) have approximately a 2-fold increased risk of cardiovascular disease (CVD) compared with women with normotensive pregnancies.^1,2,3,4,5^ Hypertensive disorders of pregnancy and CVD share common modifiable risk factors, such as adiposity, hypertension, dyslipidemia, and hyperglycemia, that may be targets for prevention. In 2018,^6^ we observed that women with HDP already had more adiposity, higher blood pressure and glucose levels, and more adverse lipid levels before first pregnancy and that their cardiovascular risk factor levels remained higher than women without HDP through age 50 years and beyond. It is not known how much of the excess CVD risk in women with history of HDP is associated with these risk factors vs how much may be caused by HDP itself or other unidentified factors. This knowledge is crucial to inform preventive action in women with a history of HDP. In a population-based cohort with longitudinal information on cardiovascular risk factors and validated information on cardiovascular events, we used mediation analysis to examine how much of the excess cardiovascular risk in women with a history of HDP is associated with adverse levels of body mass index (BMI, calculated as weight in kilograms divided by height in meters squared), blood pressure, and glucose and lipid levels.

## Methods

All procedures performed in studies involving human participants were in accordance with the ethical standards of the Regional Committee for Medical and Health Research Ethics and with the 1964 Helsinki declaration and its later amendments or comparable ethical standards. The Regional Committee for Medical and Health Research Ethics granted ethical approval of the study. In the initial Nord-Trøndelag Health Study (HUNT1), attendance and participation in questionnaires and clinical examination was considered as informed consent, and in HUNT2 and HUNT3, participants gave written consent.

### Study Population

This study included 23 885 parous women participating in the HUNT in Norway. Using the unique identification number of all Norwegian citizens, we linked information from HUNT (1984-2008), the Medical Birth Registry of Norway (MBRN, 1967-2012), the Norwegian Cause of Death Registry (1984-2015), and validated cardiovascular events from the local hospitals (1987-2015). This linked data resource has previously been used to examine the added value of pregnancy complications in clinical CVD risk prediction.^7^ See the eAppendix and eFigure in the Supplement for a description of the sample selection and an overview of the study timeline with associated data sources.

### Exposure and Covariates

Exposure was defined as history of HDP (ever HDP) in the form of preeclampsia or gestational hypertension at 40 years or younger. Additionally, we subclassified the exposure as ever preeclampsia (with or without a history of gestational hypertension) and ever gestational hypertension (but no history of preeclampsia) at 40 years or younger. Details about the diagnoses of preeclampsia and gestational hypertension in the MBRN and their validity are presented in the eAppendix of the Supplement.

We retrieved information about age at HUNT examination, self-reported ever daily smoking, highest obtained educational level, work titles, current use of antihypertensive medication, and family history of coronary heart disease (in sibling or parents) from the HUNT survey questionnaires and interviews. For 3530 women for whom educational level was not available, we deduced highest obtained educational level from their work titles based on recommendations from Statistics Norway.^8^ The MBRN provided information on mother’s age at birth and parity.

### Cardiovascular Risk Factors

Information about the most recently measured cardiovascular risk factors prior to the cardiovascular event or censoring was obtained from clinical measurements and serum samples collected at HUNT examinations. Details about the cardiovascular risk factors measurements have been reported previously^6^ and are included in the eAppendix of the Supplement.

### Cardiovascular Events

To obtain information about hospital-diagnosed cardiovascular events, medical records were retrieved for all study participants who had at least 1 record with an *International Classification of Diseases, Ninth Revision (ICD-9)* and/or *International Statistical Classification of Diseases and Related Health Problems, Tenth Revision (ICD-10) *code indicating CVD in the electronic patient administrative systems of the 2 local hospitals serving Nord-Trøndelag county between September 1, 1987, and April 24, 2015. All medical records were reviewed by 1 of 2 cardiologists (B.K. and H.D.) who, according to established criteria, confirmed any valid cardiovascular diagnoses. Additional details about the diagnoses and validation are presented in the eAppendix of the Supplement. We also obtained information on dates and causes of death up until April 24, 2015, from the Norwegian Cause of Death Registry, which has recorded all deaths in Norway since 1951.^9^ Cardiovascular disease–related deaths were identified using *ICD-9 *and *ICD-10 *codes for the underlying cause of death (eTable 1 in the Supplement).

### Statistical Analysis

We used Cox proportional hazards models^10^ to estimate the hazard ratios (HRs) for first-time cardiovascular events (fatal or nonfatal) and, specifically, first-time myocardial infarction, heart failure, and cerebrovascular events, comparing women with and without a history of HDP. We used age as the time scale, and women entered the study on September 1, 1987, their first HUNT examination, or age 40 years, whichever came last. Women were followed up until the cardiovascular event of interest, emigration from Nord-Trøndelag county, death, or April 24, 2015, whichever came first. Hazard ratios were adjusted for age (model 1) and adjusted for age, maternal birth year, highest educational level, ever daily smoking, parity before age 40 years, and family history of coronary heart disease in sibling or parents (model 2). To assess the effect of death from causes other than CVD as a competing risk, subdistribution HRs were also estimated using Fine and Gray competing risk model.^11^ The Cox proportional hazards assumption was assessed by including interactions between independent variables and time. Violations of the Cox proportional hazards assumption were handled by estimating HRs within separate age intervals in which the assumption was met.

In secondary analyses, we included only women whose first birth was recorded in the MBRN to avoid potential misclassification of women as normotensive who had earlier pregnancies not captured by the MBRN. Additionally, to avoid missing too many early cardiovascular events at younger than 40 years and owing to the complex association between parity and HDP, we examined HDP in first pregnancy as an exposure, starting exposure time at whichever came last: first birth, first HUNT participation, or September 1, 1987. In these analyses we additionally adjusted for mother’s age at first birth. Finally, because information on CVD subtypes in the Cause of Death Registry may have lower validity, we repeated the analyses using validated myocardial infarction, heart failure, and cerebrovascular events from the hospital records only.

Analogously to the study by Tanz et al,^12^ we have used a mediation approach to estimate the proportion of excess CVD risk in women with a history of HDP that is associated with conventional cardiovascular risk factors. Mediation analysis enables a decomposition of the association between exposure and outcome (called *total effect*) into a natural direct effect from exposure on outcome and a natural indirect effect from exposure on outcome through mediators.^13,14^ In our analysis, the natural indirect effect is best interpreted as the proportion of excess cardiovascular risk in women with history of HDP that is associated with conventional cardiovascular risk factors (mediators), while the natural direct effect is best understood as the proportion of excess cardiovascular risk in women who had HDP that is not associated with these factors. We estimated the part of the association between HDP and CVD that was associated with BMI, systolic and diastolic blood pressure, nonfasting serum glucose levels, and non–high-density lipoprotein (HDL) cholesterol levels (indirect effect) and the part that was not associated with these factors (direct effect) using an inverse odds ratio weighting mediation analysis method.^15,16^ A graphic and more detailed explanation of this mediation analysis is given in the Figure. Separate analyses were performed for each mediator and for the combination of BMI and blood pressure. In the mediation analysis, we additionally adjusted for the age at measurement of the mediator. Additionally, separate mediation analyses were conducted for preeclampsia and gestational hypertension as well as for CVD subtypes (myocardial infarction, heart failure, and cerebrovascular events). Mediators may have been measured before (maximum of 484 women [2%]) or after pregnancies complicated by HDP, but because we do not postulate the association between HDP and CVD to be causal and because the differences in cardiovascular risk factors between women with and without HDP are largely similar prepregnancy vs postpregnancy and throughout the age range from 20 years to older than 50 years in this study population,^6^ the timing of mediator measurement was less relevant. In 2 separate sensitivity analyses, we excluded women who had their cardiovascular risk factors measured before their first pregnancy and restricted the mediation analysis to women who had mediators measured at older than 40 years, the time where we ended exposure follow-up. All analyses were performed using Stata IC, version 14 (StataCorp).^17^ The *P *value was 2-sided, and the level of statistical significance was .05.

**Figure.**
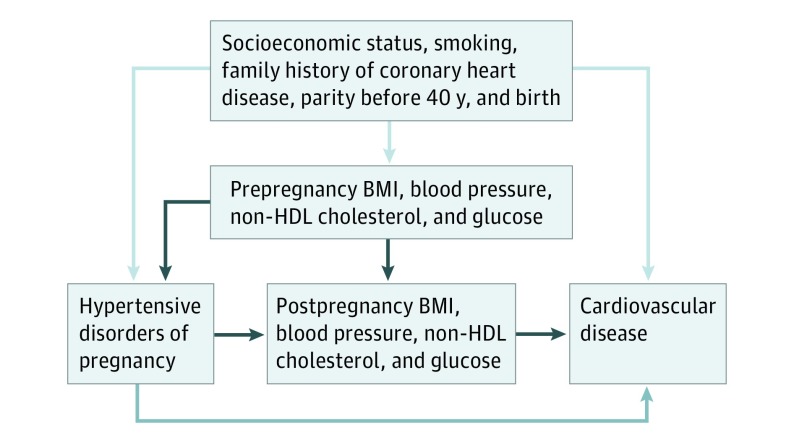
Mediation Analysis Diagram of associations between hypertensive disorders of pregnancy (HDP); cardiovascular risk factors in the form of body mass index (BMI), blood pressure, and glucose and non–high-density lipoprotein (HDL) cholesterol levels; and cardiovascular disease. The dark blue arrows indicate proportion of excess cardiovascular risk in women with HDP that is associated with BMI, blood pressure, and glucose and non-HDL cholesterol levels (indirect effect). The blue arrows indicate proportion of excess cardiovascular risk in women with HDP that is not associated with BMI, blood pressure, and glucose and non-HDL cholesterol levels (direct effect). The light blue arrows indicate confounding of the association between HDP and cardiovascular disease and that between cardiovascular risk factors and cardiovascular disease by socioeconomic status, smoking, family history of coronary heart disease, parity at younger than 40 years, and maternal birth year.

## Results

Of 23 885 women, 2119 (9%) had a history of HDP at younger than 40 years; 1391 had at least 1 occurrence of preeclampsia; and 728 experienced gestational hypertension only (Table 1). Women with history of HDP were less likely to report daily smoking and more likely to have first births captured by the MBRN than women with normotensive pregnancies (eTable 2 in the Supplement). The median ages at measurement of the cardiovascular risk factors included in the mediation analysis were 50 years for women with only normotensive pregnancies and 48 years for women with a history of HDP. Pregnancies complicated by HDP were more likely to result in preterm delivery or offspring born small for gestational age (eTable 2 in the Supplement). During a median follow-up of 18 years, 1688 women experienced at least 1 cardiovascular event, and 1565 (92.7%) had a cardiovascular event validated from hospital records. Five hundred fifty-three of 1688 women with cardiovascular events experienced a myocardial infarction, 233 had heart failure, and 878 experienced a cerebrovascular event.

**Table 1.** Descriptive Characteristics of the Study Population

Maternal Characteristic	Pregnancy Status, No. (%)^a^			
	Always Normotensive (n = 21 766)	Ever Hypertensive Disorder (n = 2119)	Gestational Hypertension Only (n = 728)	Ever Preeclampsia (n = 1391)
Birth y, median (IQR)	1954 (1946-1962)	1955 (1948-1963)	1953 (1946-1960)	1957 (1949-1964)
Age at first birth, y	24 (21-27)	23 (21-27)	23 (21-27)	24 (21-27)
Parity at younger than 40 y				
1	4907 (23)	323 (15)	113 (16)	210 (15)
2	9149 (42)	887 (42)	300 (41)	587 (42)
≥3	7710 (35)	909 (43)	315 (43)	594 (43)
First birth recorded in the MBRN				
No	4190 (19)	214 (10)	113 (16)	101 (7)
Yes	17576 (81)	1905 (90)	615 (84)	1290 (93)
Family history of coronary heart disease				
No	13991 (64)	1328 (63)	456 (63)	872 (63)
Yes	7775 (36)	791 (37)	272 (37)	519 (37)
Ever smoked daily				
No	8161 (37)	1041 (49)	336 (46)	705 (51)
Yes	13605 (63)	1078 (51)	392 (54)	686 (49)
Education				
Lower secondary	5562 (26)	517 (24)	211 (29)	306 (22)
Upper secondary	9412 (43)	949 (45)	317 (44)	632 (45)
Tertiary	6792 (31)	653 (31)	200 (27)	453 (33)
Age at measurement of cardiovascular risk factors, y, median (IQR)	50 (41-59)	48 (40-56)	51 (41-58)	46 (39-55)

Abbreviations: IQR, interquartile range; MBRN, Medical Birth Registry of Norway.

^a^ Pregnancy status designates presence of hypertensive disorder, preeclampsia, or gestational hypertension in births at younger than 40 years.

### Association Between HDP and CVD

Because the proportional hazards assumption was violated, as indicated by an interaction between the history of HDP and time (β = 0.98; 95% CI, 0.96-1.00; *P* = .01), we estimated HRs within the age intervals (40-70 years and 70-88 years) separately. For the purpose of brevity and clarity, only fully adjusted HRs based on model 2 are hereafter described in the text. Women with a history of HDP had an increased risk of any cardiovascular event (HR, 1.57; 95% CI, 1.32-1.86) between ages 40 and 70 years compared with women with only normotensive pregnancies (Table 2). The corresponding HRs were 1.66 (95% CI, 1.34-2.06) for women experiencing preeclampsia and 1.43 (95% CI, 1.09-1.88) for women experiencing gestational hypertension only. For women older than 70 years, the association was reversed, and women with a history of HDP had a lower risk of any cardiovascular event (HR, 0.60; 95% CI, 0.34-1.04) compared with women with only normotensive pregnancies. The results were broadly similar for women with preeclampsia and gestational hypertension.

**Table 2.** Hazard Ratios for Cardiovascular Events in Women With Hypertensive Disorder of Pregnancy

Event	No. of Events/No. of Women	Person-Years	Model 1, HR (95% CI)^a^	*P* Value	Model 2, HR (95% CI)^b^	*P* Value
Any CVD event						
Age (40-70 y)						
Always normotensive	1155/21 752	37 4372	1 [Reference]	NA	1 [Reference]	NA
Ever hypertensive disorder	145/2117	34 802	1.45 (1.22-1.72)	<.001	1.57 (1.32-1.86)	<.001
Ever preeclampsia	91/1389	21 714	1.52 (1.23-1.88)	<.001	1.66 (1.34-2.06)	<.001
Ever gestational hypertension	54/728	13 088	1.34 (1.02-1.76)	.04	1.43 (1.09-1.88)	.01
Age (70-88 y)						
Always normotensive	375/3499	91 989	1 [Reference]	NA	1 [Reference]	NA
Ever hypertensive disorder	13/225	5957	0.59 (0.34-1.02)	.06	0.59 (0.34-1.04)	.07
Ever preeclampsia	8/129	3406	0.70 (0.35-1.42)	.33	0.71 (0.35-1.43)	.33
Ever gestational hypertension	5/96	2551	0.46 (0.19-1.12)	.09	0.47 (0.20-1.15)	.10
Myocardial infarction						
Age (40-70 y)						
Always normotensive	383/21 752	380 698	1 [Reference]	NA	1 [Reference]	NA
Ever hypertensive disorder	54/2117	35 533	1.64 (1.23-2.18)	.001	1.86 (1.40-2.48)	<.001
Ever preeclampsia	35/1389	22 119	1.78 (1.26-2.52)	.001	2.08 (1.46-2.95)	<.001
Ever gestational hypertension	19/728	13 413	1.43 (0.90-2.26)	.13	1.56 (0.99-2.48)	.06
Age (70-88 y)						
Always normotensive	112/3499	92 782	1 [Reference]	NA	1 [Reference]	NA
Ever hypertensive disorder	4/225	5997	0.58 (0.21-1.57)	.29	0.66 (0.24-1.79)	.41
Ever preeclampsia	2/129	3437	0.57 (0.14-2.30)	.43	0.64 (0.16-2.61)	.53
Ever gestational hypertension	2/96	2560	0.59 (0.15-2.40)	.46	0.67 (0.17-2.73)	.58
Heart failure						
Age (40-70 y)						
Always normotensive	140/21 752	383 087	1 [Reference]	NA	1 [Reference]	NA
Ever hypertensive disorder	16/2117	35 850	1.47 (0.86-2.52)	.16	1.59 (0.92-2.73)	.10
Ever preeclampsia	13/1389	22 316	1.83 (0.99-3.40)	.06	2.00 (1.07-3.73)	.03
Ever gestational hypertension	6/728	13 534	0.96 (0.35-2.60)	.94	1.01 (0.37-2.75)	.97
Age (70-88 y)						
Always normotensive	73/3499	92 975	1 [Reference]	NA	1 [Reference]	NA
Ever hypertensive disorder	4/225	5994	0.87 (0.35-2.14)	.76	0.98 (0.39-2.44)	.97
Ever preeclampsia	2/129	3441	0.97 (0.31-3.06)	.96	1.07 (0.33-3.41)	.91
Ever gestational hypertension	2/96	2553	0.76 (0.19-3.07)	.70	0.87 (0.21-3.57)	.85
Cerebrovascular disease						
Age (40-70 y)						
Always normotensive	617/21 752	378 902	1 [Reference]	NA	1 [Reference]	NA
Ever hypertensive disorder	75/2117	35 324	1.40 (1.10-1.78)	.006	1.47 (1.15-1.87)	.002
Ever preeclampsia	46/1389	22 035	1.46 (1.08-1.97)	.01	1.52 (1.13-2.06)	.006
Ever gestational hypertension	29/728	13 289	1.32 (0.90-1.93)	.15	1.38 (0.95-2.02)	.10
Age (70-88 y)						
Always normotensive	178/3499	92581	1 [Reference]	NA	1 [Reference]	NA
Ever hypertensive disorder	8/225	5967	0.78 (0.40-1.52)	.47	0.75 (0.38-1.48)	.41
Ever preeclampsia	6/129	3411	0.98 (0.43-2.20)	.95	0.93 (0.41-2.10)	.86
Ever gestational hypertension	2/96	2555	0.56 (0.18-1.74)	.31	0.55 (0.18-1.73)	.31

Abbreviations: CVD, cardiovascular disease; HR, hazard ratio; NA, not applicable.

^a^ Adjusted for age.

^b^ Adjusted for age, highest obtained educational level, ever smoked daily, parity at younger than 40 years, maternal birth year, and family history of coronary heart disease.

Women with a history of HDP had an increased risk of myocardial infarction (HR, 1.86; 95% CI, 1.40-2.48), heart failure (HR, 1.59; 95% CI, 0.92-2.73), and cerebrovascular events (HR, 1.47; 95% CI, 1.15-1.87) in the age interval of 40 to 70 years compared with women with normotensive pregnancies (Table 2). These HRs were consistently higher among women with a history of preeclampsia than among women with a history of gestational hypertension only. At older than 70 years, women with a history of HDP had lower hazard rates for most subtypes of CVD compared with women with normotensive pregnancies, but limited observations and events for this age interval prevented precise estimates.

Competing risk models gave virtually identical HRs to those estimated in the main analysis (results not shown), suggesting censoring was uninformative. Sensitivity analyses restricted to women who had their first birth recorded in the MBRN also yielded similar, but slightly lower and more imprecise HRs (eTables 3-6 in the Supplement). Sensitivity analyses restricted to validated diagnoses (eTables 7 and 8 in the Supplement) gave almost identical results to the main analyses.

### Contribution of Cardiovascular Risk Factors to CVD Risk in Women With History of HDP

All associations between HDP and CVD described in this section are HRs based on the Cox proportional hazards model including person-time from 40 to 70 years. Because the associations between HDP and CVD were reversed for women older than 70 years, no mediation analyses were performed for this age interval. The associations between HDP and CVD differed slightly according to which cardiovascular risk factor was analyzed owing to variations in study population but fell within a fairly narrow range of 1.53 to 1.58 (Table 3). We calculated the percentage excess risk associated with each risk factor by dividing the β coefficient for the part of the association between HDP and CVD that was associated with the cardiovascular risk factor(s) with the β coefficient for the total association between HDP and CVD. The proportion of the association between HDP and CVD that was associated with BMI was 41%, corresponding to an HR of 1.19 (95% CI, 1.07-1.33). Systolic and diastolic blood pressure was associated with 60% and 73% of the association between HDP and CVD, corresponding to HRs of 1.30 (95% CI, 1.16-1.47) and 1.38 (95% CI, 1.23-1.55), respectively. Combining BMI with systolic and diastolic pressure in 2 separate mediation analyses showed that BMI together with systolic and diastolic blood pressure was associated with 67% and 79% of the excess cardiovascular risk in women with history of HDP, respectively (Table 3).

**Table 3.** Association Between Hypertensive Pregnancy Disorders and Cardiovascular Disease and BMI, Blood Pressure, and Serum Glucose and Lipid Levels in Women Aged 40 to 70 Years

Cardiovascular Risk Factors	Women, No.	Total Association Between HDP and CVD		Part of Association Between HDP and CVD That Is Not Associated With the Examined Cardiovascular Risk Factors		Part of Association Between HDP and CVD That Is Associated With the Examined Cardiovascular Risk Factors		Proportion of Excess Cardiovascular Risk in Women Who Had HDP That Is Associated With Cardiovascular Risk Factor(s), %^b^
		HR (95% CI)^a^	*P* Value	HR (95% CI)^a^	*P* Value	HR (95% CI)^a^	*P* Value	
BMI	23 508	1.54 (1.29-1.83)	<.001	1.29 (1.06-1.56)	.01	1.19 (1.07-1.33)	.001	41
Systolic blood pressure	23 500	1.55 (1.29-1.86)	<.001	1.19 (0.97-1.47)	.10	1.30 (1.16-1.47)	<.001	60
Diastolic blood pressure	23 501	1.55 (1.30-1.85)	<.001	1.13 (0.92-1.38)	.25	1.38 (1.23-1.55)	<.001	73
Glucose	21 881	1.58 (1.30-1.92)	<.001	1.40 (1.15-1.72)	.001	1.12 (1.02-1.23)	.01	25
Non-HDL cholesterol	21 517	1.53 (1.26-1.88)	<.001	1.38 (1.12-1.69)	.002	1.11 (1.02-1.21)	.02	24
BMI and systolic blood pressure	23 453	1.53 (1.27-1.84)	<.001	1.15 (0.91-1.44)	.23	1.33 (1.16-1.53)	<.001	67
BMI and diastolic blood pressure	23 454	1.53 (1.28-1.83)	<.001	1.09 (0.88-1.36)	.43	1.40 (1.22-1.61)	<.001	79

Abbreviations: BMI, body mass index (calculated as weight in kilograms divided by height in meters squared); CVD, cardiovascular disease; HDL, high-density lipoprotein; HDP, hypertensive disorders of pregnancy.

^a^ Estimates are adjusted for age, highest obtained educational level, ever smoked daily, parity at younger than age 40 years, maternal birth year, and family history of coronary heart disease.

^b^ We calculated the percentage excess risk associated with each risk factor by dividing the β coefficient for the part of the association between HDP and CVD that was associated with the cardiovascular risk factor(s) with the β coefficient for the total association between HDP and CVD.

We had fewer observations of nonfasting glucose levels and non-HDL cholesterol levels because these risk factors were not routinely assessed in HUNT1. Glucose was associated with 25% and non-HDL cholesterol was associated with 24%, corresponding to HRs of 1.12 (95% CI, 1.02-1.23) and 1.11 (95% CI, 1.02-1.21), respectively (Table 3). All risk factors combined (BMI, blood pressure, glucose, and non-HDL cholesterol) did not have a greater association with excess cardiovascular risk than the combination of BMI and blood pressure (results not shown).

Separate mediation analyses for history of preeclampsia and gestational hypertension suggested that blood pressure had a greater association with cardiovascular risk in women with gestational hypertension, where it was associated with all excess risk. In women with preeclampsia, the mediators were maximally associated with 79% of the excess risk (eTables 9 and 10 in the Supplement). Analyses of CVD subtypes indicated that blood pressure was associated with most of the excess risk of heart failure and cerebrovascular events in women with a history of HDP, but only up to 41% of the excess risk of myocardial infarction (eTables 11-13 in the Supplement). Excluding the 484 women who had their cardiovascular risk factors measured before first pregnancy did not substantially alter our results (results not shown). Among the approximately 18 000 women who had their cardiovascular risk factors measured at older than 40 years, the proportions of excess CVD risk in women with a history of HDP that was associated with the cardiovascular risk factors was moderately reduced compared with the overall study population and maximally were associated with 47% of the excess risk (eTable 14 in the Supplement).

## Discussion

In this population-based cohort study, women with a history of HDP had approximately 60% higher risk of CVD until age 70 years compared with women with normotensive pregnancies. About 79% of the excess CVD risk was associated with blood pressure and BMI, indicating that these risk factors are important targets for CVD prevention in these women. The relative risk of CVD was slightly larger for women who experienced preeclampsia compared with gestational hypertension and higher for myocardial infarction than for heart failure and cerebrovascular events. The proportion of excess CVD risk associated with blood pressure and BMI was moderately lower among women who had their cardiovascular risk factors measured at older than 40 years, suggesting that earlier measurements of cardiovascular risk factors is more informative about later CVD risk in women with history of HDP.

To our knowledge, no previous mediation analysis has combined measurements of cardiovascular risk factors with validated cardiovascular events to examine this topic, but our results are supported by an abstract from the Nurses’ Health Study II^18^ showing that self-reported cardiovascular risk factors, in particular chronic hypertension, were associated with most of the excess CVD risk associated with HDP. Information on which modifiable factors explain the excess CVD risk in women with history of HDP is a key requisite to inform prevention of CVD in these women. Previous large Nordic studies^19,20,21,22,23,24^ have used data from national health registries to quantify the association between HDP and future CVD. However, some of them included fatal events only,^21,22^ few studies examined the risk of heart failure and cerebrovascular events, and none had measurements of cardiovascular risk factors to perform mediation analyses.

Our estimates of the associations between HDP and future CVD are generally consistent with those of previous studies,^1,2,3,4,5,19^ but our point estimates are on the lower end of the spectrum.^1,2,3,4,5,19,20^ Most studies and meta-analyses report a doubling in CVD risk for preeclampsia,^1,2,3,4^ but previous cohort studies^19,20,21,22,23,24^ in comparable study populations reported associations that are relatively similar to our results. For example, in a nationwide Norwegian study,^20^ preeclampsia was associated with an HR of 1.6 for CVD mortality and an HR of 2.1 for major coronary events, and gestational hypertension was associated with an HR of 1.8 for CVD.^19^ In a Swedish study population, women with gestational hypertension and mild and severe preeclampsia had relative risks of ischemic heart disease of 1.6, 1.9, and 2.8, respectively.^23^ Similar estimates for various CVD end points were observed in a Danish population.^24^ Fewer studies have examined the associations of HDP with heart failure and cerebrovascular events, but a history of preeclampsia was associated with a 3.6-fold increased risk of heart failure in a meta-analysis.^2^ Meta-analyses of the association between a history of preeclampsia and cerebrovascular disease have reported an HR of 2.0^4^ and an odds ratio of 1.8.^1^ Most previous studies followed up the women from the time of pregnancy, whereas we could not follow up women between the start of the MBRN in 1967 and the introduction of electronic hospital records in 1987. In this younger age group, the relative risk of CVD in women with a history of HDP may be higher (even if the absolute risk is low), which could explain the lower HRs observed in our study. Although we did not have statistical power to make conclusive inferences about the association between HDP and CVD among women older than 70 years, the apparent reversal of the HRs at old age is similar to what has been observed between cardiovascular risk factors and mortality in elderly populations^25^ and may be a result of survivor bias.

### Strengths and Limitations

Our study was, with a median follow-up of 18 years, longer than the follow-up reported in the other Nordic studies.^19,20,21,22,23^ We started follow-up time after women largely finished reproducing at age 40 years to avoid introducing immortal time bias.^26^ Compared with most other studies relying on registry or administrative event data only, our study had the advantage of having clinically measured information about conventional cardiovascular risk factors and having validated 93% of the cardiovascular outcomes, thus ensuring high specificity of the outcome variables. However, we acknowledge that the available tests for CVD have changed throughout our study period and that this could potentially have affected our estimates. We also acknowledge that we may have missed nonfatal events where the patient was not admitted to hospital, but owing to the excellent public access to health care in Norway throughout the study period, this number is expectedly very low. Also, any misclassification would expectedly not depend on HDP or the examined mediators, and we consider it unlikely that this may have substantially influenced our results. Additionally, we had access to a broad range of relevant confounders from the HUNT study, enabling analyses of the association of these cardiovascular risk factors with the excess cardiovascular risk in women with history of HDP. For this purpose, we used a novel approach to mediation analysis^15^ that allowed us to perform formal mediation analyses for single and joint effects of several cardiovascular risk factors on the association between HDP and CVD in a survival setting while adjusting for multiple confounders. Our mediation results are probably generalizable to other populations where, as in Norway, access to health care is free and clinical follow-up is generally good. However, the association of these cardiovascular risk factors with later CVD risk may be lower in our and similar populations compared with populations where no or little medical treatment of cardiovascular risk factors takes place, ie, in populations where health care access is more limited.

## Conclusions

Our study has shown that women with history of HDP have an increased risk of CVD that is to a large extent associated with increased levels of conventional, modifiable cardiovascular risk factors. Blood pressure plays a substantial role in driving the excess cardiovascular risk in women who experienced preeclampsia and an even larger role in women who experienced gestational hypertension. The association of conventional risk factors, in particular blood pressure and BMI, with the development of CVD in women with history HDP indicate that preventive efforts aimed at decreasing the levels of these risk factors could reduce cardiovascular risk in women with history of HDP.

## Notes

Supplement. **eAppendix.** **eTable 1. **ICD Codes for Fatal Cardiovascular Events in the Cause of Death Registry **eFigure.** Timeline of Follow-Up With Data Sources **eTable 2. **Descriptive Characteristics of Included Pregnancies in Main Analysis **eTable 3.** Hazard Ratios (HRs) For Any CVD Event and Myocardial Infarction in women With Hypertensive Disorder of Pregnancy and Whose First Birth Was Recorded in the Medical Birth Registry of Norway **eTable 4.** Hazard ratios (HRs) For Heart Failure and Cerebrovascular Disease Events in Women With Hypertensive Disorder of Pregnancy and Whose First Birth was Recorded in the Medical Birth Registry of Norway. **eTable 5.** Hazard ratios (HRs) For Any CVD Event and Myocardial Infarction in Women With Hypertensive Disorder in Their First Pregnancy **eTable 6**. Hazard ratios (HRs) For Heart Failure and Cerebrovascular Disease in Women With Hypertensive Disorder in Their First Pregnancy **eTable 7.** Hazard Ratios for Any Validated CVD Event and Validated Myocardial Infarction in Women With Hypertensive Disorder of Pregnancy **eTable 8.** Hazard Ratios For Validated Heart Failure and Cerebrovascular Disease Events in Women With Hypertensive Disorder Of Pregnancy **eTable 9.** Association Between Preeclampsia and Cardiovascular Disease Decomposed into Parts Not Explained by and Explained by BMI, Blood Pressure, Glucose and Lipids on Cardiovascular Disease in Women **eTable 10.** Association Between Gestational Hypertension and Cardiovascular Disease Decomposed into Parts Not Explained by and Explained by BMI, Blood Pressure, Glucose And Lipids on Cardiovascular Disease in Women **eTable 11.** Association Between Hypertensive Pregnancy Disorders and myocardial Infarction Decomposed into Parts Not Explained by and Explained by Through BMI, Blood Pressure, Glucose and Lipids in Women **eTable 12.** Association Between Hypertensive Pregnancy Disorders and Heart Failure Decomposed into Parts Not Explained by and Explained by BMI, Blood Pressure, Glucose and Lipids in Women **eTable 13.** Association Between Hypertensive Pregnancy Disorders and Cerebrovascular Disease Decomposed into Parts Not Explained by and Explained by BMI, Blood Pressure, Glucose and Lipids on Cerebrovascular Disease in Women **eTable 14.** Association Between Hypertensive Pregnancy Disorders and Cardiovascular Disease Decomposed into Parts Not Explained by and Explained by BMI, Blood Pressure, Serum Glucose and Lipids Measured After Age 40 in Women
