# Supplementary material for: Association of Conventional Cardiovascular Risk Factors With Cardiovascular Disease After Hypertensive Disorders of Pregnancy: Analysis of the Nord-Trøndelag Health Study
Source: JAMA Cardiol. 2019 Jun 12;4(7):628–35. doi: 10.1001/jamacardio.2019.1746 (PMC6563586; doi:10.1001/jamacardio.2019.1746)
Supplement: Supplement. — eAppendix. eTable 1. ICD Codes for Fatal Cardiovascular Events in the Cause of Death Registry eFigure. Timeline of Follow-Up With Data Sources eTable 2. Descriptive Characteristics of Included Pregnancies in Main Analysis eTable 3. Hazard Ratios (HRs) For Any CVD Event and Myocardial Infarction in women With Hypertensive Disorder of Pregnancy and Whose First Birth Was Recorded in the Medical Birth Registry of Norway eTable 4. Hazard ratios (HRs) For Heart Failure and Cerebrovascular Disease Events in Women With Hypertensive Disorder of Pregnancy and Whose First Birth was Recorded in the Medical Birth Registry of Norway. eTable 5. Hazard ratios (HRs) For Any CVD Event and Myocardial Infarction in Women With Hypertensive Disorder in Their First Pregnancy eTable 6. Hazard ratios (HRs) For Heart Failure and Cerebrovascular Disease in Women With Hypertensive Disorder in Their First Pregnancy eTable 7. Hazard Ratios for Any Validated CVD Event and Validated Myocardial Infarction in Women With Hypertensive Disorder of Pregnancy eTable 8. Hazard Ratios For Validated Heart Failure and Cerebrovascular Disease Events in Women With Hypertensive Disorder Of Pregnancy eTable 9. Association Between Preeclampsia and Cardiovascular Disease Decomposed into Parts Not Explained by and Explained by BMI, Blood Pressure, Glucose and Lipids on Cardiovascular Disease in Women eTable 10. Association Between Gestational Hypertension and Cardiovascular Disease Decomposed into Parts Not Explained by and Explained by BMI, Blood Pressure, Glucose And Lipids on Cardiovascular Disease in Women eTable 11. Association Between Hypertensive Pregnancy Disorders and myocardial Infarction Decomposed into Parts Not Explained by and Explained by Through BMI, Blood Pressure, Glucose and Lipids in Women eTable 12. Association Between Hypertensive Pregnancy Disorders and Heart Failure Decomposed into Parts Not Explained by and Explained by BMI, Blood Pressure, Glucose and Lipids in Women eTable 13. Association [file jamacardiol-4-628-s001.pdf]

Supplementary Online Content

Huag EB, Horn J, Markovitz AR, et al. Association of conventional cardiovascular risk factors with cardiovascular disease after hypertensive disorders of pregnancy: analysis of the Nord-Trøndelag Health Study. Published online June 12, 2019. *JAMA Cardiol*. doi:10.1001/jamacardio.2019.1746

eAppendix.

eTable 1. ICD Codes for Fatal Cardiovascular Events in the Cause of Death Registry

eFigure. Timeline of Follow-Up With Data Sources

eTable 2. Descriptive Characteristics of Included Pregnancies in Main Analysis

eTable 3. Hazard Ratios (HRs) For Any CVD Event and Myocardial Infarction in women With Hypertensive Disorder of Pregnancy and Whose First Birth Was Recorded in the Medical Birth Registry of Norway

eTable 4. Hazard ratios (HRs) For Heart Failure and Cerebrovascular Disease Events in Women With Hypertensive Disorder of Pregnancy and Whose First Birth was Recorded in the Medical Birth Registry of Norway.

eTable 5. Hazard ratios (HRs) For Any CVD Event and Myocardial Infarction in Women With Hypertensive Disorder in Their First Pregnancy

eTable 6. Hazard ratios (HRs) For Heart Failure and Cerebrovascular Disease in Women With Hypertensive Disorder in Their First Pregnancy

eTable 7. Hazard Ratios for Any Validated CVD Event and Validated Myocardial Infarction in Women With Hypertensive Disorder of Pregnancy

eTable 8. Hazard Ratios For Validated Heart Failure and Cerebrovascular Disease Events in Women With Hypertensive Disorder Of Pregnancy

eTable 9. Association Between Preeclampsia and Cardiovascular Disease Decomposed into Parts Not Explained by and Explained by BMI, Blood Pressure, Glucose and Lipids on Cardiovascular Disease in Women

eTable 10. Association Between Gestational Hypertension and Cardiovascular Disease Decomposed into Parts Not Explained by and Explained by BMI, Blood Pressure, Glucose And Lipids on Cardiovascular Disease in Women

eTable 11. Association Between Hypertensive Pregnancy Disorders and myocardial Infarction Decomposed into Parts Not Explained by and Explained by Through BMI, Blood Pressure, Glucose and Lipids in Women

eTable 12. Association Between Hypertensive Pregnancy Disorders and Heart Failure Decomposed into Parts Not Explained by and Explained by BMI, Blood Pressure, Glucose and Lipids in Women

© 2019 Haug EB et al. *JAMA Cardiology.*

eTable 13. Association Between Hypertensive Pregnancy Disorders and Cerebrovascular Disease Decomposed into Parts Not Explained by and Explained by BMI, Blood Pressure, Glucose and Lipids on Cerebrovascular Disease in Women

eTable 14. Association Between Hypertensive Pregnancy Disorders and Cardiovascular Disease Decomposed into Parts Not Explained by and Explained by BMI, Blood Pressure, Serum Glucose and Lipids Measured After Age 40 in Women

This supplementary material has been provided by the authors to give readers additional information about their work.

© 2019 Haug EB et al. *JAMA Cardiology.*

Study Population

HUNT is an ongoing longitudinal population-based study that since the 1980s has invited all residents aged 20 years and above of Nord-Trøndelag county in Norway to undergo comprehensive health assessments with clinical measurements, blood sampling, questionnaires and interviews. To date, three surveys have been conducted; HUNT1[1] 1984-86, HUNT2 1995-97[2] and HUNT3 2006-08[3]. Participation rates for women were 89.9% in HUNT1[1], 75.5% in HUNT2[2] and 58.7% in HUNT3[3]. The population in Nord-Trøndelag county is predominantly White and considered to be representative of Norway as a whole[2].

MBRN[4] has recorded all births in a mandatory registry since 1967 and contains information on child and maternal health including details of pregnancy complications. In total, 31 364 women from HUNT had at least one birth registered in MBRN between 1967 and 2012. Since we defined our exposure based on history of pregnancy complications by age 40, we excluded 454 women who had their first birth after age 40 and 3901 women who turned 40 after 31st December 2012, the end of the MBRN follow-up. We excluded 3133 births (and consequently 227 women) which either 1) were multiple pregnancies, 2) had a gestational length < 20 weeks, 3) were preceded by maternal chronic hypertension, 4) produced offspring with a birth weight <350 grams or 5) lacked information on birth weight and gestational length. Further, 1593 women were excluded due to incomplete information on smoking, educational level or family history of coronary heart disease. Additionally, we excluded 1012 women who moved out of Nord-Trøndelag county and 292 women with cardiovascular events before start of follow-up, resulting in a final study population of 23 885 women.

Exposure

Diagnoses of preeclampsia and gestational hypertension in the MBRN were based on internationally recommended diagnostic criteria[5]: Gestational hypertension was generally defined as *de novo* hypertension (≥140 mmHg systolic and/or ≥90 mmHg diastolic) after 20 weeks of gestation, and preeclampsia also required proteinuria (300 mg/24 hours or ≥1+ on the dipstick test). Previous studies[6,7] have validated the MBRN diagnoses of preeclampsia and gestational hypertension within the HUNT study population finding predictive positive values of 88% and 68% for preeclampsia and gestational hypertension, respectively.

Cardiovascular risk factors

All serum analyses were performed in fresh non-fasting samples at the Central Laboratory, Levanger Hospital, Nord-Trøndelag Hospital Trust using a Hitachi 911 Autoanalyzer in HUNT2 and Architect cSystems ci8200 in HUNT3. Height and weight were measured with the person wearing light clothes and no shoes and were rounded to the nearest cm (height) and half kilo (weight). BMI was calculated as weight (in kg) divided by the squared value of height (in m). Blood pressure in HUNT1 was measured manually two times at 1-minute intervals using a sphygmomanometer after the person had come to rest, and we used the mean value of these two measurements in our analysis. In HUNT2 and HUNT3, blood pressure was measured three times at 1-minute intervals using an automatic oscillometric method (Dinamap, Critikon, Florida) after the person had come to rest, with cuff size adjusted to arm circumference. We used the mean of the second and third measurement, except for 2153 women in HUNT3 who lacked the third measurement due to sick leave amongst staff; for them, we used the second measurement only. Serum total and high density lipoprotein (HDL) cholesterol and triglycerides were analyzed using enzymatic colorimetric methods (Boeheringer Mannheim, Germany) in HUNT2. In HUNT3 HDL cholesterol was measured with an accelerator selective detergent methodology, total cholesterol was analyzed by a cholesterol esterase methodology by equipment from Abbott, Clinical Chemistry, USA. Non-HDL cholesterol was calculated as the difference between total and HDL cholesterol. In HUNT1 capillary glucose was measured at the examination stations in participants above 40 years (Reflocheck-Glucose, Boehringer Mannheim, Germany), and for the analysis of mean glucose levels, we transformed capillary levels to equate serum values (in mmol/L) by

multiplying with 1.11[8]. In HUNT2 and HUNT3 serum glucose was measured for all persons using an enzymatic hexokinase method.

Supplemental Validation Information

From the electronic patient administrative system at the two primary hospitals in Nord-Trøndelag county, Levanger Hospital and Namsos Hospital (Nord-Trøndelag Hospital Trust), we obtained information on all study participants registered with at least one of the following cardiovascular diagnoses between September 1, 1987 (the first date of electronic recording) and April 24, 2015: ICD-9,402, 404, 410-414, 425, 427.5, 428, 430-438, 440-448; ICD-10, G45, I11, I13, I20-I25, I42, I46, I50, I60-I67, I71, I72, I74. For each identified patient, one of two experienced cardiologists (B.K., H.D.), who were unaware of the pregnancy history of the participants, examined the medical records to determine the first validated occurrence of each of the following cardiovascular events: myocardial infarction, heart failure, cardiac arrest, cerebral infarction, transient ischaemic attack (TIA), intracranial hemorrhage, aortic dissection or aneurysm, dissection or aneurysm of peripheral arteries, and embolism or thrombosis in peripheral arteries. Myocardial infarction diagnosed using ESC/ACCF/AHA/WHF criteria[9] and classified as STEMI or NSTEMI. Heart failure was diagnosed based on echocardiography findings combined with symptoms/signs according to ESC guidelines,[10] and was classified as systolic or diastolic. Ejection fraction was recorded or, if possible, estimated based on the echocardiography description in cases where no value was reported. If echocardiography had not been performed, the diagnosis was made using the clinically based Framingham criteria.[11] Right ventricular failure without left ventricular failure (e.g. cor pulmonale following pulmonary embolism or COPD) was not included. Cardiac arrest was diagnosed based on a documented or highly probable cardial cause of pulselessness and lack of breathing requiring cardiopulmonary resuscitation at hospital or before hospital admission. Cerebral infarction was diagnosed based on typical symptoms and signs combined with radiological evidence from computer tomography (CT) or magnetic resonance imaging (MRI) scans. In some cases, the diagnosis of cerebral infarction was based on typical symptoms and signs in absence of radiological evidence, but only if CT or MRI scan had been performed, and no alternative explanation for the clinical presentation was found. TIA was diagnosed based on typical symptoms/signs combined with absence of radiological signs of cerebral infarction on CT or MRI scan. Events probably caused by other diseases were excluded. TIA occurring after intracerebral infarction was not recorded. Possible cerebrovascular events accompanying intracerebral malignancy, infection or inflammation were not included. Transitory global amnesia and migraine with aura were not considered as valid cerebrovascular diagnoses. Intracranial hemorrhage was classified based on radiological evidence as subarachnoid, intracerebral, subdural or epidural, and hemorrhage secondary to trauma or intracerebral malignancy was not included. For a diagnosis of aortic aneurysm, an aortic diameter >30 mm in abdomen or >40 mm i thorax was required.

References:

1. Holmen J, Midthjell K, Bjartveit K, Hjort PF, Lund-Larsen PG. *The Nord-Trøndelag Health Survey 1984-1986. Purpose, Background and Methods. Participation, Non-Participation and Frequency Distributions.* Verdal: Senter for samfunnsmedisinsk forskning, Statens Institutt for folkehelse(SIFF). Helsetjenesteforskning; 1990:1-257. https://www.ntnu.no/c/document_library/get_file?uuid=4be1f10f-be02-484d-a033-6194748bd5f4&groupId=10304. Accessed September 1, 2018.

2. Holmen J, Midthjell K, Krüger Ø, et al. The Nord-Trøndelag Health Study 1995-97(HUNT2). Objectives contents, methods and participation. *J Epid.* 2003;13(1):19-32.

3. Krokstad S, Langhammer A, Hveem K, et al. Cohort Profile: the HUNT Study, Norway. *Int J Epidemiol.* 2013;42(4):968-977. doi:10.1093/ije/dys095

4. Irgens LM. The Medical Birth Registry of Norway. Epidemiological research and surveillance throughout 30 years. *Acta Obstet Gynecol Scand.* 2001;79(6):435-439. doi:10.1034/j.1600-0412.2000.079006435.x

5. American College of Obstetricians and Gynecologists. Hypertension in pregnancy. Report of the American College of Obstetricians and Gynecologists' Task Force on Hypertension in Pregnancy. *Obstet Gynecol*. 2013;122(5):1122-1131. doi:10.1097/01.AOG.0000437382.03963.88

6. Thomsen LCV, Klungsøyr K, Roten LT, et al. Validity of the diagnosis of pre-eclampsia in the Medical Birth Registry of Norway. *Acta Obstet Gynecol Scand*. 2013;92(8):943-950. doi:10.1111/aogs.12159

7. Moth FN, Sebastian TR, Horn J, Rich-Edwards J, Romundstad PR, Åsvold BO. Validity of a selection of pregnancy complications in the Medical Birth Registry of Norway. *Acta Obstet Gynecol Scand*. 2016;95(5):519-527. doi:10.1111/aogs.12868

8. Sacks DB, Arnold M, Bakris GL, et al. Guidelines and Recommendations for Laboratory Analysis in the Diagnosis and Management of Diabetes Mellitus. *Diabetes Care*. 2011;34(6):e61-e99. doi:10.2337/dc11-9998

9. Thygesen K, Alpert JS, Jaffe AS, et al. Third universal definition of myocardial infarction. *Eur Heart J*. 2012;33(20):2551-2567. doi:10.1093/eurheartj/ehs184

10. McMurray JJV, Adamopoulos S, Anker SD, et al. ESC Guidelines for the diagnosis and treatment of acute and chronic heart failure 2012: The Task Force for the Diagnosis and Treatment of Acute and Chronic Heart Failure 2012 of the European Society of Cardiology. Developed in collaboration with the Heart Failure Association (HFA) of the ESC. *Eur Heart J*. 2012;33(14):1787-1847. doi:10.1093/eurheartj/ehs104

11. McKee PA, Castelli WP, McNamara PM, Kannel WB. The natural history of congestive heart failure: the Framingham study. *N Engl J Med*. 1971;285(26):1441-1446. doi:10.1056/NEJM197112232852601

eTable 1. ICD codes for fatal cardiovascular events in the Cause of Death Registry

	**ICD-9 codes (1986-95)**	**ICD-10 codes (from 1996)**
**All cardiovascular events**	401-414 and 424-445	G45, I10-I25, I34-I37, I42-I51, and I60-I77
**Myocardial infarction**	410 and 412	I21, I22 and I25.2
**Heart failure**	425 and 428	I42 and I50
**Cerebrovascular disease**	430, 431 and 433-435	G45, I60, I61, I63, I64, and I69.0, .1, .3, and .4.

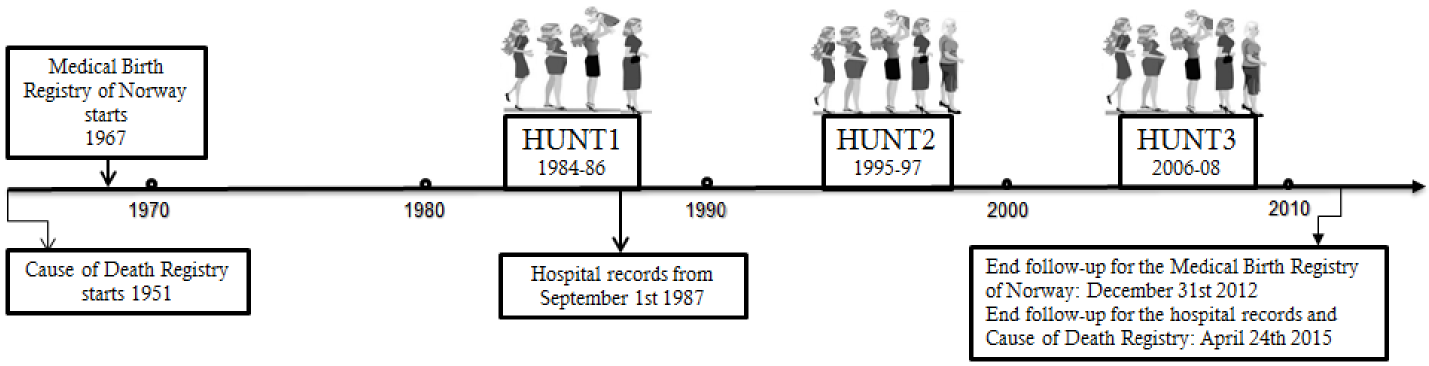

eFigure . Timeline of follow-up with data sources.

eTable 2. Descriptive characteristics of included pregnancies in main analysis

	Pregnancy status			
	**Normotensive**	**Hypertensive disorder**	**Gestational Hypertension**	**Preeclampsia**
**Number, (%)**	51155 (95)	2460 (5)	954 (2)	1506 (3)
**Gestational length in weeks, n (%)**				
<34	790 (2)	92 (4)	12 (1)	80 (5)
34-36	1478 (3)	151 (6)	22 (2)	129 (9)
≥37	46066 (90)	2062 (84)	871 (91)	1191 (79)
Missing	2821 (6)	155 (6)	49 (5)	106 (7)
**Stillbirths, n (%)**	425 (1)	38 (2)	10 (1)	28 (2)
**Birth weight*, n (%)**				
Small for gestational age	1628 (3)	195 (8)	49 (5)	146 (10)
Normal for gestational age	45424 (89)	2046 (83)	829 (87)	1217 (81)
Large for gestational age	998 (2)	55 (2)	21 (2)	34 (2)
Missing	3105 (6)	164 (7)	55 (6)	109 (7)

*Small and large for gestational age were defined as more than two standard deviations away from the established mean birth weights by gestational age in the Medical Birth Registry of Norway.[26]

eTable 3. Hazard ratios (HRs) for any CVD event and myocardial infarction in women with hypertensive disorder of pregnancy and whose first birth was recorded in the Medical Birth Registry of Norway.

				Model 1*		Model 2†	
	**No. of women**	**Person-years**	**No. of events**	**Hazard ratio (95% CI)**	**p-value**	**Hazard ratio (95% CI)**	**p-value**
**Any CVD event**							
Age [40-70]							
Always normotensive	17570	277252	706	Ref.		Ref.	
Ever hypertensive disorder	1904	30141	104	1.35 (1.10 , 1.66)	0.004	1.43 (1.17 , 1.76)	0.001
Ever preeclampsia	1289	19498	71	1.46 (1.14 , 1.87)	0.002	1.56 (1.22 , 2.00)	<0.001
Ever gestational hypertension	615	10643	33	1.16 (0.82 , 1.64)	0.405	1.22 (0.86 , 1.73)	0.270
Age (70-88]							
Always normotensive	799	21263	39	Ref.		Ref.	
Ever hypertensive disorder	104	2785	2	0.32 (0.08 , 1.34)	0.119	0.28 (0.06 , 1.23)	0.092
Ever preeclampsia	73	1945	2	0.46 (0.11 , 1.96)	0.294	0.39 (0.09 , 1.70)	0.208
Ever gestational hypertension	31	839	0	N/A		N/A	
**Myocardial infarction**							
Age [40-70]							
Always normotensive	17570	280282	223	Ref.		Ref.	
Ever hypertensive disorder	1904	30671	34	1.38 (0.96 , 1.99)	0.078	1.53 (1.06 , 2.19)	0.022
Ever preeclampsia	1289	19827	24	1.55 (1.02 , 2.36)	0.041	1.73 (1.13 , 2.64)	0.011
Ever gestational hypertension	615	10844	10	1.10 (0.58 , 2.07)	0.770	1.19 (0.63 , 2.24)	0.596
Age (70-88]							
Always normotensive	799	21344	11	Ref.		Ref.	
Ever hypertensive disorder	104	2786	1	0.55 (0.07 , 4.25)	0.566	0.61 (0.07 , 5.05)	0.643
Ever preeclampsia	73	1947	1	0.79 (0.10 , 6.11)	0.820	0.79 (0.09 , 6.60)	0.824
Ever gestational hypertension	31	839	0	N/A		N/A	

*Adjusted for age. †Adjusted for age, maternal birth year, highest obtained educational level, ever smoked daily, age at first birth, parity before age 40 and family history of coronary heart disease. CVD=cardiovascular disease.

eTable 4. Hazard ratios (HRs) for heart failure and cerebrovascular disease events in women with hypertensive disorder of pregnancy and whose first birth was recorded in the Medical Birth Registry of Norway.

				Model 1*		Model 2†	
	No. of women	Person-years	No. of events	Hazard ratio (95% CI)	p-value	Hazard ratio (95% CI)	p-value
**Heart failure**							
Age [40-70]							
Always normotensive	17570	281214	70	Ref.		Ref.	
Ever hypertensive disorder	1904	30804	7	0.94 (0.43 , 2.04)	0.869	0.98 (0.45 , 2.15)	0.962
Ever preeclampsia	1289	19909	5	1.06 (0.43 , 2.63)	0.902	1.12 (0.45 , 2.78)	0.811
Ever gestational hypertension	615	10895	2	0.73 (0.18 , 2.97)	0.657	0.75 (0.18 , 3.07)	0.689
Age (70-88]							
Always normotensive	799	21351	8	Ref.		Ref.	
Ever hypertensive disorder	104	2786	1	0.61 (0.08 , 4.71)	0.633	1.25 (0.14 , 11.00)	0.838
Ever preeclampsia	73	1947	1	0.87 (0.11 , 6.75)	0.890	1.61 (0.19 , 13.56)	0.662
Ever gestational hypertension	31	839	0	N/A		N/A	
**Cerebrovascular disease**							
Age [40-70]							
Always normotensive	17570	279102	378	Ref.		Ref.	
Ever hypertensive disorder	1904	30395	60	1.46 (1.11 , 1.92)	0.007	1.52 (1.15 , 2.00)	0.003
Ever preeclampsia	1289	19672	41	1.58 (1.14 , 2.18)	0.005	1.65 (1.19 , 2.27)	0.003
Ever gestational hypertension	615	10723	19	1.25 (0.79 , 1.99)	0.335	1.30 (0.82 , 2.06)	0.267
Age (70-88]							
Always normotensive	799	21322	18	Ref.		Ref.	
Ever hypertensive disorder	104	2788	1	0.32 (0.04 , 2.44)	0.269	0.26 (0.03 , 2.08)	0.205
Ever preeclampsia	73	1949	1	0.45 (0.06 , 3.50)	0.445	0.36 (0.05 , 2.87)	0.334
Ever gestational hypertension	31	839	0	N/A		N/A	

*Adjusted for age. †Adjusted for age, highest obtained educational level, ever smoked daily, age at first birth, parity before age 40, maternal birth year and family history of coronary heart disease.

ETable 5. Hazard ratios (HRs) for any CVD event and myocardial infarction in women with hypertensive disorder in their first pregnancy.

				Model 1*		Model 2†	
	**No. of women**	**Person-years**	**No. of events**	**Hazard ratio (95% CI)**	**p-value**	**Hazard ratio (95% CI)**	**p-value**
**Any CVD event**							
Age [22-70]							
First pregnancy							
Normotensive	18617	397595	767	Ref.		Ref.	
Hypertensive disorder	1277	26903	70	1.39 (1.09 , 1.77)	0.009	1.49 (1.16 , 1.90)	0.002
Preeclampsia	873	17886	48	1.48 (1.11 , 1.98)	0.008	1.61 (1.20 , 2.16)	0.001
Gestational hypertension	404	17886	22	1.22 (0.80 , 1.86)	0.360	1.27 (0.83 , 1.95)	0.264
Age (70-88]							
First pregnancy							
Normotensive	815	21688	39	Ref.		Ref.	
Hypertensive disorder	80	2148	2	0.37 (0.09 , 1.56)	0.174	0.29 (0.06 , 1.27)	0.100
Preeclampsia	57	1520	2	0.53 (0.12 , 2.28)	0.398	0.41 (0.09 , 1.84)	0.245
Gestational hypertension	23	1520	0	N/A		N/A	
**Myocardial infarction**							
Age [22-70]							
First pregnancy							
Normotensive	18617	401158	231	Ref.		Ref.	
Hypertensive disorder	1277	27208	24	1.57 (1.03 , 2.39)	0.035	1.79 (1.17 , 2.73)	0.007
Preeclampsia	873	18100	15	1.53 (0.91 , 2.58)	0.112	1.79 (1.06 , 3.02)	0.030
Gestational hypertension	404	18100	9	1.65 (0.85 , 3.22)	0.139	1.79 (0.92 , 3.49)	0.087
Age (70-88]							
First pregnancy							
Normotensive	815	21768	11	Ref.		Ref.	
Hypertensive disorder	80	2150	1	0.63 (0.08 , 4.91)	0.661	0.63 (0.07 , 5.27)	0.668
Preeclampsia	57	1522	1	0.91 (0.12 , 7.13)	0.930	0.79 (0.09 , 6.74)	0.832
Gestational hypertension	23	1522	0	N/A		N/A	

*Adjusted for age. †Adjusted for age, highest obtained educational level, ever smoked daily, age at first birth, maternal birth year and family history of coronary heart disease. CVD=cardiovascular disease.

eTable 6. Hazard ratios (HRs) for heart failure and cerebrovascular disease in women with hypertensive disorder in their first pregnancy.

				Model 1*		Model 2†	
	**No. of women**	**Person-years**	**No. of events**	**Hazard ratio (95% CI)**	**p-value**	**Hazard ratio (95% CI)**	**p-value**
**Heart failure**							
Age [22-70]							
First pregnancy							
Normotensive	18617	402101	74	Ref.		Ref.	
Hypertensive disorder	1277	27336	5	1.05 (0.42 , 2.59)	0.922	1.09 (0.44 , 2.72)	0.848
Preeclampsia	873	18159	4	1.30 (0.47 , 3.55)	0.613	1.37 (0.50 , 3.77)	0.541
Gestational hypertension	404	18159	1	0.59 (0.08 , 4.24)	0.600	0.60 (0.08 , 4.36)	0.617
Age (70-88]							
First pregnancy							
Normotensive	815	21776	8	Ref.		Ref.	
Hypertensive disorder	80	2150	1	0.71 (0.09 , 5.51)	0.739	1.44 (0.16 , 12.62)	0.741
Preeclampsia	57	1522	1	1.01 (0.13 , 7.92)	0.993	1.81 (0.21 , 15.28)	0.585
Gestational hypertension	23	1522	0	N/A		N/A	
**Cerebrovascular disease**							
Age [22-70]							
First pregnancy							
Normotensive	18617	399554	421	Ref.		Ref.	
Hypertensive disorder	1277	27100	42	1.52 (1.11 , 2.09)	0.010	1.58 (1.15 , 2.18)	0.005
Preeclampsia	873	17994	31	1.75 (1.21 , 2.52)	0.003	1.83 (1.27 , 2.64)	0.001
Gestational hypertension	404	17994	11	1.11 (0.61 , 2.02)	0.733	1.14 (0.63 , 2.08)	0.667
Age (70-88]							
First pregnancy							
Normotensive	815	21746	18	Ref.		Ref.	
Hypertensive disorder	80	2152	1	0.37 (0.05 , 2.92)	0.348	0.28 (0.03 , 2.24)	0.227
Preeclampsia	57	1524	1	0.53 (0.07 , 4.21)	0.550	0.40 (0.05 , 3.28)	0.390
Gestational hypertension	23	1524	0	N/A		N/A	

*Adjusted for age. †Adjusted for age, highest obtained educational level, ever smoked daily, age at first birth, maternal birth year and family history of coronary heart disease.

eTable 7. Hazard ratios for any validated CVD event and validated myocardial infarction in women with hypertensive disorder of pregnancy.									
					Model 1*			Model 2†	
	**No. of women**	**Person-years**	**No. of events**		**Hazard ratio (95% CI)**	**p-value**		**Hazard ratio (95% CI)**	**p-value**
**Any CVD event**									
Age [40-70]									
Always normotensive	21752	374372	1074		Ref.			Ref.	
Ever hypertensive disorder	2117	34802	138		1.48 (1.24 , 1.77)	<0.001		1.60 (1.34 , 1.92)	<0.001
Ever preeclampsia	1389	21714	87		1.56 (1.26 , 1.94)	<0.001		1.70 (1.37 , 2.12)	<0.001
Ever gestational hypertension	728	13088	51		1.36 (1.03 , 1.80)	0.031		1.45 (1.10 , 1.93)	0.009
Age (70-88]									
Always normotensive	3499	91989	341		Ref.			Ref.	
Ever hypertensive disorder	225	5957	12		0.60 (0.34 , 1.06)	0.079		0.60 (0.34 , 1.08)	0.087
Ever preeclampsia	129	3406	8		0.77 (0.38 , 1.56)	0.476		0.77 (0.38 , 1.55)	0.460
Ever gestational hypertension	96	2551	4		0.41 (0.15 , 1.09)	0.075		0.42 (0.16 , 1.14)	0.088
**Myocardial infarction**									
Age [40-70]									
Always normotensive	21752	380698	383		Ref.			Ref.	
Ever hypertensive disorder	2117	35533	54		1.64 (1.23 , 2.18)	0.001		1.86 (1.40 , 2.48)	<0.001
Ever preeclampsia	1389	22119	35		1.78 (1.26 , 2.52)	0.001		2.08 (1.46 , 2.95)	<0.001
Ever gestational hypertension	728	13413	19		1.43 (0.90 , 2.26)	0.131		1.56 (0.99 , 2.48)	0.058
Age (70-88]									
Always normotensive	3499	92782	112		Ref.			Ref.	
Ever hypertensive disorder	225	5997	4		0.58 (0.21 , 1.57)	0.285		0.66 (0.24 , 1.79)	0.410
Ever preeclampsia	129	3437	2		0.57 (0.14 , 2.30)	0.429		0.64 (0.16 , 2.61)	0.534
Ever gestational hypertension	96	2560	2		0.59 (0.15 , 2.40)	0.464		0.67 (0.17 , 2.73)	0.578

*Adjusted for age. †Adjusted for age, highest obtained educational level, ever smoked daily, parity before age 40, maternal birth year and family history of coronary heart disease.

**eTable 8. Hazard ratios for validated heart failure and cerebrovascular disease events in women with hypertensive disorder of pregnancy.**									
					**Model 1***			**Model 2†**	
	**No. of women**	**Person-years**	**No. of events**		**Hazard ratio (95% CI)**	**p-value**		**Hazard ratio (95% CI)**	**p-value**
**Heart failure**									
Age [40-70]									
Always normotensive	21752	383087	138		Ref.			Ref.	
Ever hypertensive disorder	2117	35850	16		1.49 (0.87 , 2.55)	0.148		1.60 (0.93 , 2.75)	0.090
Ever preeclampsia	1389	22316	12		1.85 (0.99 , 3.43)	0.052		2.02 (1.08 , 3.77)	0.027
Ever gestational hypertension	728	13534	4		0.97 (0.36 , 2.63)	0.951		1.02 (0.38 , 2.77)	0.966
Age (70-88]									
Always normotensive	3499	92975	72		Ref.			Ref.	
Ever hypertensive disorder	225	5994	4		0.89 (0.36 , 2.19)	0.799		1.00 (0.40 , 2.49)	0.996
Ever preeclampsia	129	3441	2		0.99 (0.31 , 3.13)	0.986		1.09 (0.34 , 3.48)	0.887
Ever gestational hypertension	96	2553	2		0.77 (0.19 , 3.13)	0.717		0.90 (0.22 , 3.67)	0.881
**Cerebrovascular disease**									
Age [40-70]									
Always normotensive	21752	378902	598		Ref.			Ref.	
Ever hypertensive disorder	2117	35324	73		1.43 (1.12 , 1.82)	0.004		1.49 (1.17 , 1.91)	0.001
Ever preeclampsia	1389	22035	45		1.47 (1.09 , 2.00)	0.012		1.54 (1.13 , 2.08)	0.006
Ever gestational hypertension	728	13289	28		1.36 (0.93 , 1.99)	0.111		1.43 (0.98 , 2.09)	0.067
Age (70-88]									
Always normotensive	3499	92581	175		Ref.			Ref.	
Ever hypertensive disorder	225	5967	8		0.71 (0.35 , 1.43)	0.333		0.68 (0.33 , 1.38)	0.281
Ever preeclampsia	129	3411	6		0.99 (0.44 , 2.24)	0.986		0.93 (0.41 , 2.12)	0.868
Ever gestational hypertension	96	2555	2		0.38 (0.09 , 1.52)	0.170		0.37 (0.09 , 1.50)	0.163

*Adjusted for age. †Adjusted for age, highest obtained educational level, ever smoked daily, age at first birth, parity before age 40, maternal birth year and family history of coronary heart disease.

eTable 9. Association between preeclampsia and cardiovascular disease decomposed into parts not explained by and explained by BMI, blood pressure, glucose and lipids on cardiovascular disease in women aged [40-70].

	No. of women	Total association between preeclampsia and CVD[*]		Part of association between preeclampsia and CVD that is not explained by the examined cardiovascular risk factors[†]		Part of association between preeclampsia and CVD that is explained by the examined cardiovascular risk factor(s)[‡]		Proportion excess cardiovascular risk in women who had preeclampsia that is explained by cardiovascular risk factor(s)
**Mediator (s)**		**HR[§] (95% CI)**	**p-value**	**HR[§] (95% CI)**	**p-value**	**HR[§] (95% CI)**	**p-value**	**Percentage**
**BMI**	22790	1.63 (1.31 , 2.03)	<0.001	1.45 (1.10 , 1.92)	0.008	1.12 (0.93 , 1.36)	0.242	23
**Systolic blood pressure**	22781	1.66 (1.33 , 2.05)	<0.001	1.41 (1.04 , 1.92)	0.028	1.17 (0.95 , 1.45)	0.144	32
**Diastolic blood pressure**	22782	1.63 (1.30 , 2.06)	<0.001	1.19 (0.92 , 1.56)	0.189	1.37 (1.18 , 1.58)	<0.001	64
**Glucose**	21227	1.69 (1.35 , 2.13)	<0.001	1.53 (1.20 , 1.96)	0.001	1.10 (0.98 , 1.25)	0.117	19
**Non-HDL cholesterol**	20881	1.63 (1.28 , 2.08)	<0.001	1.45 (1.13 , 1.87)	0.004	1.12 (1.00 , 1.25)	0.056	23
**BMI and systolic blood pressure**	22735	1.60 (1.27 , 2.00)	<0.001	1.22 (0.93 , 1.59)	0.148	1.31 (1.11 , 1.54)	0.001	58
**BMI and diastolic blood pressure**	22736	1.60 (1.28 , 2.00)	<0.001	1.10 (0.85 , 1.44)	0.469	1.45 (1.23 , 1.70)	0.000	79

CVD=cardiovascular disease, HR=hazard ratio; CI=confidence interval; BMI=body mass index; HDL=high density lipoprotein.

[*]Corresponds to the total effect of HDP on CVD in mediation analysis terminology. [†]Corresponds to the direct effect of HDP on CVD in mediation analysis terminology. [‡]Corresponds to the direct effect of HDP on CVD in mediation analysis terminology. [§]Estimates are adjusted for age, maternal birth year, highest obtained educational level, ever smoked daily, parity before age 40 and family history of coronary heart disease.

eTable 10. Association between gestational hypertension and cardiovascular disease decomposed into parts not explained by and explained by BMI, blood pressure, glucose and lipids on cardiovascular disease in women aged [40-70].							
	No. of women	Total association between gestational hypertension and CVD*		Part of association between gestational hypertension and CVD that is not explained by the examined cardiovascular risk factors†		Part of association between gestational hypertension and CVD that is explained by the examined cardiovascular risk factor(s)‡		Proportion excess cardiovascular risk in women who had gestational hypertension that is explained by cardiovascular risk factor(s)¶
**Mediator (s)**		**HR§ (95% CI)**	**p-value**	**HR§ (95% CI)**	**p-value**	**HR§ (95% CI)**	**p-value**	**Percentage**
**BMI**	22148	1.44 (1.09 , 1.90)	0.010	1.25 (0.90 , 1.73)	0.180	1.15 (0.96 , 1.39)	0.127	39
**Systolic blood pressure**	22141	1.44 (1.09 , 1.89)	0.009	0.97 (0.68 , 1.37)	0.843	1.49 (1.18 , 1.89)	0.001	110
**Diastolic blood pressure**	22142	1.44 (1.09 , 1.90)	0.011	0.99 (0.70 , 1.42)	0.971	1.45 (1.15 , 1.82)	0.002	102
**Glucose**	20624	1.40 (1.03 , 1.91)	0.030	1.23 (0.90 , 1.69)	0.192	1.14 (0.98 , 1.32)	0.079	38
**Non-HDL cholesterol**	20274	1.40 (1.03 , 1.91)	0.033	1.26 (0.91 , 1.74)	0.161	1.11 (0.96 , 1.28)	0.158	31
**BMI and systolic blood pressure**	22098	1.43 (1.09 , 1.88)	0.009	1.02 (0.72 , 1.46)	0.895	1.40 (1.10 , 1.78)	0.006	93
**BMI and diastolic blood pressure**	22099	1.44 (1.10 , 1.88)	0.007	1.04 (0.73 , 1.47)	0.835	1.39 (1.09 , 1.77)	0.008	90

HR=hazard ratio; CI=confidence interval; BMI=body mass index; HDL=high density lipoprotein.
*Corresponds to the total effect of HDP on CVD in mediation analysis terminology. †Corresponds to the direct effect of HDP on CVD in mediation analysis terminology.
‡Corresponds to the direct effect of HDP on CVD in mediation analysis terminology. §Estimates are adjusted for age, maternal birth year, highest obtained educational level, ever smoked daily, parity before age 40 and family history of coronary heart disease. ¶The proportion mediated will exceed 100% when the direct effect of HDP go below 1 and appear protective.

eTable 11. Association between hypertensive pregnancy disorders and myocardial infarction decomposed into parts not explained by and explained by through BMI, blood pressure, glucose and lipids in women aged [40-70].							
	No. of women	Total association between HDP and CVD*		Part of association between HDP and CVD that is not explained by the examined cardiovascular risk factors†		Part of association between HDP and CVD that is explained by the examined cardiovascular risk factor(s)‡		Proportion excess cardiovascular risk in women who had HDP that is explained by cardiovascular risk factor(s)
Mediator(s)		HR§ (95% CI)	p-value	HR§ (95% CI)	p-value	HR§ (95% CI)	p-value	Percentage
BMI	23508	1.71 (1.29 , 2.28)	<0.001	1.53 (1.11 , 2.12)	0.009	1.12 (0.96 , 1.30)	0.155	20
Systolic blood pressure	23500	1.69 (1.26 , 2.25)	<0.001	1.42 (1.01 , 2.00)	0.042	1.19 (0.99 , 1.42)	0.065	33
Diastolic blood pressure	23501	1.69 (1.27 , 2.24)	<0.001	1.39 (1.02 , 1.91)	0.038	1.21 (1.03 , 1.42)	0.017	37
Glucose	21881	1.82 (1.34 , 2.48)	<0.001	1.75 (1.26 , 2.42)	0.001	1.04 (0.92 , 1.18)	0.496	7
Non-HDL cholesterol	21517	1.69 (1.20 , 2.38)	0.002	1.52 (1.07 , 2.17)	0.002	1.11 (0.98 , 1.26)	0.094	20
BMI and systolic blood pressure	23453	1.68 (1.26 , 2.25)	<0.001	1.39 (0.99 , 1.95)	0.057	1.21 (0.98 , 1.48)	0.070	36
BMI and diastolic blood pressure	23454	1.68 (1.27 , 2.23)	<0.001	1.36 (0.97 , 1.91)	0.076	1.24 (1.02 , 1.49)	0.030	41

HDP=hypertensive disorders of pregnancy, CVD=cardiovascular disease, HR=hazard ratio; CI=confidence interval; BMI=body mass index; HDL=high density lipoprotein.
*Corresponds to the total effect of HDP on CVD in mediation analysis terminology. †Corresponds to the direct effect of HDP on CVD in mediation analysis terminology.
‡Corresponds to the direct effect of HDP on CVD in mediation analysis terminology. §Estimates are adjusted for age, highest obtained educational level, ever smoked daily, parity before age 40, maternal birth year and family history of coronary heart disease.

eTable 12. Association between hypertensive pregnancy disorders and heart failure decomposed into parts not explained by and explained by BMI, blood pressure, glucose and lipids in women aged [40-70].								
	No. of women	Total association between HDP and CVD*		Part of association between HDP and CVD that is not explained by the examined cardiovascular risk factors†		Part of association between HDP and CVD that is explained by the examined cardiovascular risk factor(s)‡		Proportion excess cardiovascular risk in women who had HDP that is explained by cardiovascular risk factor(s)¶
**Mediator(s)**		**HR§ (95% CI)**	**p-value**	**HR§ (95% CI)**	**p-value**	**HR§ (95% CI)**	**p-value**	**Percentage**
**BMI**	23508	1.40 (0.76 , 2.60)	0.283	0.92 (0.43 , 1.97)	0.830	1.52 (1.02 , 2.27)	0.038	125
**Systolic blood pressure**	23500	1.51 (0.84 , 2.69)	0.164	1.37 (0.64 , 2.94)	0.414	1.10 (0.69 , 1.74)	0.690	23
**Diastolic blood pressure**	23501	1.51 (0.83 , 2.75)	0.179	1.02 (0.45 , 2.30)	0.959	1.48 (0.88 , 2.47)	0.137	95
**Glucose**	21881	1.63 (0.88 , 3.01)	0.122	1.28 (0.65 , 2.51)	0.479	1.27 (0.98 , 1.65)	0.071	50
**Non-HDL cholesterol**	21517	1.50 (0.78 , 2.89)	0.229	1.26 (0.61 , 2.59)	0.528	1.19 (0.90 , 1.56)	0.222	43
**BMI and systolic blood pressure**	23453	1.40 (0.75 , 2.62)	0.292	1.09 (0.45 , 2.62)	0.849	1.28 (0.74 , 2.24)	0.377	75
**BMI and diastolic blood pressure**	23454	1.40 (0.79 , 2.49)	0.251	0.79 (0.33 , 1.89)	0.602	1.76 (0.95 , 3.26)	0.070	169

HDP=hypertensive disorders of pregnancy, CVD=cardiovascular disease, HR=hazard ratio; CI=confidence interval; BMI=body mass index; HDL=high density lipoprotein.
*Corresponds to the total effect of HDP on CVD in mediation analysis terminology. †Corresponds to the direct effect of HDP on CVD in mediation analysis terminology.
‡Corresponds to the direct effect of HDP on CVD in mediation analysis terminology. §Estimates are adjusted for age, highest obtained educational level, ever smoked daily, parity before age 40, maternal birth year and family history of coronary heart disease. ¶The proportion mediated will exceed 100% when the direct effect of HDP go below 1 and appear protective.

eTable 13. Association between hypertensive pregnancy disorders and cerebrovascular disease decomposed into parts not explained by and explained by BMI, blood pressure, glucose and lipids on cerebrovascular disease in women aged [40-70].

	No. of women	Total association between HDP and CVD[*]		Part of association between HDP and CVD that is not explained by the examined cardiovascular risk factors [†]		Part of association between HDP and CVD that is explained by the examined cardiovascular risk factor(s)[‡]		Proportion excess cardiovascular risk in women who had HDP that is explained by cardiovascular risk factor(s)
**Mediator(s)**		**HR[§] (95% CI)**	**p-value**	**HR[§] (95% CI)**	**p-value**	**HR[§] (95% CI)**	**p-value**	**Percentage**
**BMI**	23508	1.44 (1.13 , 1.83)	0.003	1.24 (0.94 , 1.62)	0.123	1.16 (1.01 , 1.33)	0.030	41
**Systolic blood pressure**	23500	1.47 (1.14 , 1.89)	0.003	1.07 (0.79 , 1.46)	0.647	1.36 (1.15 , 1.63)	0.001	81
**Diastolic blood pressure**	23501	1.47 (1.13 , 1.91)	0.004	1.04 (0.77 , 1.42)	0.782	1.40 (1.20 , 1.65)	0.000	89
**Glucose**	21881	1.42 (1.07 , 1.88)	0.014	1.27 (0.94 , 1.70)	0.116	1.12 (1.00 , 1.25)	0.043	33
**Non-HDL cholesterol**	21517	1.50 (1.16 , 1.94)	0.002	1.34 (1.03 , 1.75)	0.031	1.12 (1.01 , 1.23)	0.033	27
**BMI and systolic blood pressure**	23453	1.45 (1.13 , 1.86)	0.004	1.08 (0.78 , 1.48)	0.651	1.34 (1.10 , 1.64)	0.004	80
**BMI and diastolic blood pressure**	23454	1.45 (1.14 , 1.84)	0.003	1.06 (0.79 , 1.43)	0.704	1.36 (1.13 , 1.65)	0.001	84

HDP=hypertensive disorders of pregnancy, CVD=cardiovascular disease, HR=hazard ratio; CI=confidence interval; BMI=body mass index; HDL=high density lipoprotein.
[*]Corresponds to the total effect of HDP on CVD in mediation analysis terminology. [†]Corresponds to the direct effect of HDP on CVD in mediation analysis terminology.
[‡]Corresponds to the direct effect of HDP on CVD in mediation analysis terminology. [§]Estimates are adjusted for age, highest obtained educational level, ever smoked daily, parity before age 40, maternal birth year and family history of coronary heart disease.

eTable 14. Association between hypertensive pregnancy disorders and cardiovascular disease decomposed into parts not explained by and explained by BMI, blood pressure, serum glucose and lipids measured after age 40 in women aged [40-70].

	No. of women	Total association between HDP and CVD[*]		Part of association between HDP and CVD that is not explained by the examined cardiovascular risk factors [†]		Part of association between HDP and CVD that is explained by the examined cardiovascular risk factor(s)[‡]		Proportion excess cardiovascular risk in women who had HDP that is explained by cardiovascular risk factor(s)
**Mediator(s)**		**HR[§] (95% CI)**	**p-value**	**HR[§] (95% CI)**	**p-value**	**HR[§] (95% CI)**	**p-value**	**Percentage**
**BMI**	18034	1.56 (1.29 , 1.90)	<0.001	1.37 (1.11 , 1.69)	0.003	1.14 (1.01 , 1.29)	0.038	29
**Systolic blood pressure**	18029	1.58 (1.30 , 1.92)	<0.001	1.27 (1.02 , 1.59)	0.033	1.24 (1.09 , 1.42)	0.002	47
**Diastolic blood pressure**	18031	1.58 (1.30 , 1.93)	<0.001	1.28 (1.04 , 1.59)	0.023	1.23 (1.09 , 1.40)	0.001	46
**Glucose**	17643	1.58 (1.29 , 1.93)	<0.001	1.40 (1.14 , 1.72)	0.001	1.13 (1.02 , 1.25)	0.020	26
**Non-HDL cholesterol**	17280	1.53 (1.24 , 1.88)	<0.001	1.40 (1.14 , 1.72)	0.002	1.09 (0.99 , 1.20)	0.075	21
**BMI and systolic blood pressure**	17990	1.56 (1.27 , 1.90)	<0.001	1.25 (0.99 , 1.58)	0.066	1.24 (1.06 , 1.46)	0.006	50
**BMI and diastolic blood pressure**	17992	1.56 (1.28 , 1.89)	<0.001	1.26 (1.01 , 1.59)	0.042	1.23 (1.06 , 1.43)	0.007	47

HDP=hypertensive disorders of pregnancy, CVD=cardiovascular disease, HR=hazard ratio; CI=confidence interval; BMI=body mass index; HDL=high density lipoprotein.
[*]Corresponds to the total effect of HDP on CVD in mediation analysis terminology. [†]Corresponds to the direct effect of HDP on CVD in mediation analysis terminology.
[‡]Corresponds to the direct effect of HDP on CVD in mediation analysis terminology. [§]Estimates are adjusted for age, highest obtained educational level, ever smoked daily, parity before age 40, maternal birth year and family history of coronary heart disease.
